# Surveillance and characteristics of food-borne outbreaks in the Netherlands, 2006 to 2019

**DOI:** 10.2807/1560-7917.ES.2022.27.3.2100071

**Published:** 2022-01-20

**Authors:** Ingrid HM Friesema, Ife A Slegers-Fitz-James, Ben Wit, Eelco Franz

**Affiliations:** 1 Epidemiology and Surveillance of Infectious Diseases, Centre for Infectious Disease Control, National Institute for Public Health and the Environment (RIVM), Bilthoven, the Netherlands; 2 Dutch Food and Consumer Product Safety Authority (NVWA), Utrecht, the Netherlands

**Keywords:** food-borne outbreaks, surveillance, norovirus, Salmonella, Campylobacter

## Abstract

**Background:**

A wide variety of pathogens can cause disease in humans via consumption of contaminated food. Although food-borne outbreaks only account for a small part of the food-borne disease burden, outbreak surveillance can provide insights about the pathogens, food products implied as vehicle, points of contamination, and the settings in which transmission occurs.

**Aim:**

To describe the characteristics of food-borne outbreaks registered between 2006 and 2019 in the Netherlands.

**Methods:**

All reported outbreaks in which the first case occurred during 2006–19 were analysed. We examined the number of outbreaks, cases and setting by year, aetiology, type of evidence and food commodities.

**Results:**

In total, 5,657 food-borne outbreaks with 27,711 cases were identified. The contaminated food product could be confirmed in 152 outbreaks (2.7%); in 514 outbreaks (9.1%), a pathogen was detected in cases and/or environmental swabs. Norovirus caused most outbreaks (205/666) and most related cases (4,436/9,532), followed by *Salmonella* spp. (188 outbreaks; 3,323 cases) and *Campylobacter* spp. (150 outbreaks; 601 cases). *Bacillus cereus* was most often found in outbreaks with a confirmed food vehicle (38/152). Additionally, a connection was seen between some pathogens and food commodities. Public eating places were most often mentioned as a setting where the food implicated in the outbreak was prepared.

**Conclusion:**

Long-term analysis of food-borne outbreaks confirms a persistent occurrence. Control and elimination of food-borne illness is complicated since multiple pathogens can cause illness via a vast array of food products and, in the majority of the outbreaks, the pathogen remains unknown.

## Introduction

A wide variety of pathogens can cause disease in humans through the consumption of contaminated food [1-3]. Contamination of food can occur at any point from farm to table, as a result of improper hygiene, handling, storage or preparation, and the broad range of food products that can be contaminated adds to the complexity. An estimated 652,000 cases of infectious diseases because of contaminated food occurred in 2018 in the Netherlands, leading to around EUR 171 million in costs [4]. This figure and corresponding costs have remained at the same level since 2009 [4,5]. The exact number of cases remains unknown; only a minority of food-borne cases is captured by surveillance systems since most infections are relatively mild and no diagnostic testing is performed. Furthermore, not all food-borne infections are systematically monitored. 

Although recognised food-borne outbreaks only account for a small part of the food-borne disease burden, they can provide insight into the pathogens causing outbreaks, food products implied as vehicles, points of contamination, and settings in which transmission occurs [6,7]. Determination of the contaminated food product is difficult, especially in sporadic cases, because of varying incubation periods in which many exposures occurred, as well as recall bias. Outbreaks offer the opportunity to gather consumption data from more than one case and to perform a comparison with controls, which increases the chance of finding the contaminated food item. Analysis of data over a longer period also offers the opportunity to describe trends in food-borne outbreaks, to identify new and emerging food-borne pathogens and specific pathogen-food combinations, and to examine the public health importance of pathogens, which can be used to improve food safety [6,8].

The aim of this study is to describe the characteristics of food-borne outbreaks registered between 2006 and 2019 in the Netherlands in order to provide a better understanding of food-borne outbreaks and to guide efforts to control, reduce and prevent future food-borne illness.

## Methods

### Surveillance systems for food-borne outbreaks in the Netherlands

In the Netherlands, three main surveillance pathways for the detection of food-borne outbreaks exist.

Firstly, professionals, such as doctors and microbiologists, mandatorily report outbreaks to the regional public health service (PHS) when there are two or more human cases with the same disease and/or infection, and with a probable link to the same food source. The PHS investigates the outbreak, often together with the Dutch Food Safety Authority (Nederlandse Voedsel en Waren Autoriteit (NVWA)). Each PHS notifies all food-borne outbreaks to the National Institute for Public Health and the Environment (Rijksinstituut voor Volksgezondheid en Milieu (RIVM)) as soon as possible. The 25 PHS report these outbreaks via a digital notification system that went online in 2003. 

Secondly, outbreaks can be detected via sequence data gathered within the national disease-specific surveillance systems for *Salmonella*, *Listeria monocytogenes*, Shiga toxin-producing *Escherichia coli* (STEC) and hepatitis A. With such events, the RIVM coordinates and analyses the outbreak on behalf of all PHS. Also, outbreaks detected simultaneously in more than one PHS region can shift to this pathway. Since 2012, national outbreaks have been manually added by the RIVM to the same digital notification system as used by the PHS.

Thirdly, a pathway exists for citizens who suspect to have become ill following consumption of food or drinks. Citizens can report directly to the NVWA, which is recorded in a standardised form. When sufficient information is given, the NVWA will investigate the possible food-borne outbreak. The NVWA has used the same online system as the PHS since 2006 to report outbreaks to the RIVM, although the system does not facilitate joint reports. Up to 2014, all outbreaks in which food samples or environmental swabs were taken were reported. These criteria were broadened in 2015 to all non-anonymous reports of outbreaks, regardless whether food/environmental samples were taken. Basic laboratory investigations were typically performed for suspected bacterial contamination. If other contamination were suspected, like the presence of viruses or scombroid toxins, these analyses were added. However, results of virus investigations, both in food and environmental swabs, were mainly registered in a different laboratory system within the NVWA, which were not standardly added to the reports. Since 2012, efforts have increased to also report the virus results of these outbreaks. 

For our analyses, all outbreaks reported through these three pathways in which the first case occurred during 2006–19 were combined into one database, where paired reports of one outbreak were merged.

### Additional data on food-borne outbreaks

A parallel literature search of publications and grey literature, as well as internal databases held at RIVM, revealed some outbreaks that were not reported through one of the surveillance pathways. The largest group of missing outbreaks were national outbreaks occurring before 2012. 

### Outbreak classification

The type of evidence leading to the suspicion of the food vehicle was determined. If no pathogen could be established in cases or food, the outbreak was categorised as ‘not confirmed’. When environmental swabs were taken and tested positive for norovirus or rotavirus, and no foods tested positive, the outbreak was categorised as ‘positive environmental swabs’, regardless of whether cases also tested positive. Outbreaks for which a pathogen was identified in cases, were categorised as ‘confirmed in case(s)’, in the absence of another indicator. An outbreak was considered to have a confirmed food vehicle when a pathogen was detected in a food product or a pathogen was found in cases, in conjunction with strong epidemiological evidence from the outbreak investigation implying one food product. 

If *Bacillus cereus*, *Staphylococcus aureus* or *Clostridium perfringens* were detected, the number of colony forming units (cfu) should exceed the legal standard of 100,000 cfu per gram or millilitre food before considered harmful [9]. A cut-off of 100 cfu was used for *Listeria monocytogenes* [10]. Detections below the limits were registered and analysed as ‘not confirmed’. 

We calculated the number of outbreaks, cases, and counts per setting by aetiology and type of evidence. We also examined the number of outbreaks and cases per year for *Campylobacter* spp., *Salmonella* spp. and norovirus. All food products implicated in the confirmed food-borne outbreaks were assigned to one of 11 food commodities, i.e. fish, shellfish, red meat (pork or beef), poultry, eggs, dairy, fruit/vegetables, cereals/pasta/rice, pastry/cookies, soup/sauce, and composed products (dishes with a mixture of ingredients, for example vegetables, meat, and/or rice); food commodity–pathogen pairs were examined. 

### Analyses

The data from the three surveillance pathways entered in the digital notification system was extracted into Microsoft Excel and all additional food-borne outbreaks found in the literature search were added to the Excel database. Analyses were done using SAS 9.4 (SAS Institute, Cary, North Carolina, United States (US)). 

### Ethical statement

Ethical approval was not needed as all information gathered in the database are aggregated on outbreak level and do not contain data on individuals.

## Results

### Outbreak overview

**Figure 1:**
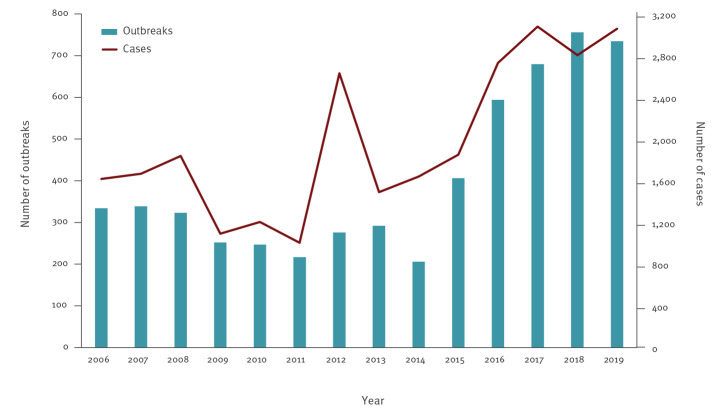
Number of food-borne outbreaks (n = 5,657) and related human cases (n = 27,711) per year, the Netherlands, 2006–2019

During 2006–19, a total of 5,657 food-borne outbreaks with 27,711 cases were identified. An average of 404 (range: 206–756) outbreaks with 1,979 (range: 1,006–3,080) cases was reported each year (Figure 1). The mean number of cases per outbreak was 4.9 (range: 1–1,149). Most outbreaks were reported to the NVWA (5,367 outbreaks and 25,659 cases) compared with PHS notifications (523 outbreaks and 8,844 cases). One outbreak consisted of a single Dutch case as part of an international outbreak. 

Data is composed of the outbreaks reported by PHS, detected through disease-specific national surveillance systems, reported by the NVWA, and the outbreaks found via the additional search. Outbreaks reported via more than one pathway are combined into one outbreak.

Within the PHS notifications, a decrease in reports was observed between 2006–12 (40–49 outbreaks/year) and 2013–19 (27–38 outbreaks/year) without a change in criteria or other clear reason. In contrast, the number of registered outbreaks by the NVWA increased substantially after changing the criteria in 2015, i.e. from 251 (range: 185–313) outbreaks per year in 2006–14 to 622 (range: 398–736) outbreaks per year in 2015–19. However, the percentage of outbreaks registered by the NVWA in which a pathogen was reported dropped from 10.7% in 2006–14 to 5.3% in 2015–19.

In total, 290 outbreaks were only reported as a PHS notification, 5,134 only via the NVWA, and 233 in both systems. In the PHS-only notifications, a pathogen was detected in 86.6% (n = 251) of cases, 0.7% (n = 2) in food/environment, or both in 1.7% (n = 5). In NVWA-only reports, a pathogen was detected in 1.2% (n = 62), 3.2% (n = 164), and 0.2% (n = 11), respectively, for cases, food/environment or both. Finally, the distribution within outbreaks reported by both institutions was 41.2% (n = 96), 8.6% (n = 20), and 23.6% (n = 55). Strong epidemiological evidence existed in nine PHS notifications (3.1%) and in 14 outbreaks reported by both (6.0%). A positive food product was found in seven PHS notifications (2.4%), 89 NVWA-only reports (1.7%) and 33 outbreaks reported by both (14.2%). 

Differences were observed based on the setting of the outbreak. In PHS notifications (n = 290), private home (n = 102; 35.2%), infected abroad (n = 70; 24.1%) and restaurant/deli/cafeteria (n = 51; 17.6%) are most mentioned, whereas restaurant/deli/cafeteria (n = 4,181; 81.4%), plant/facility (n = 370; 7.2%) and private home (n = 250; 4.9%) are most mentioned in NVWA-only reports (n = 5,134), and restaurant/deli/cafeteria (n = 2; 48.1%), catering (n = 37; 15.9%) and plant/facility (n = 30; 12.9%) in outbreaks reported by both (n = 233).

### Aetiology

In 666 (11.8%) of the 5,657 outbreaks, a pathogen was reported to be detected in food, the environment and/or in cases (Table 1). The food product causing the outbreak could be confirmed in 152 outbreaks (22.8%); in the remaining 514 outbreaks (77.2%), a pathogen was only detected in cases and/or environmental swabs. The mean number of cases per outbreak with a confirmed food vehicle was 23.4 (range: 2–1,149) cases, compared with 22.5 (range: 2–150) cases with positive environmental swabs, 8.0 (range: 1–195) cases with a pathogen detected in patients, and 3.6 (range: 2–160) cases in outbreaks without a pathogen identified.

**Table 1:** Number and percentage of food-borne outbreaks (n = 5,657) and related human cases (n = 27,711), by aetiology and confirmation of food vehicle, the Netherlands, 2006–2019

Aetiology	Outbreaks (n = 5,657)					Cases (n = 27,711)					
	Confirmed food vehicle	Other	Total			Confirmed food vehicle	Other	Total			
			n	%	Outbreaks per year (range)			n	%	Cases per outbreak	
										Mean	Range
***Bacillus cereus***	**33**	**2**	**35**	**0.6**	**0–6**	**227**	**7**	**234**	**0.8**	**7**	**2–90**
***Clostridium spp./C. botulinum***	**0**	**2**	**2**	**0.04**	**0–1**	**0**	**11**	**11**	**0.04**	**6**	**3–8**
***Clostridium perfringens***	**5**	**0**	**5**	**0.1**	**0–2**	**192**	**0**	**192**	**0.7**	**38**	**2–180**
***Staphylococcus aureus***	**11**	**1**	**12**	**0.2**	**0–5**	**62**	**55**	**117**	**0.4**	**10**	**2–55**
***Campylobacter* spp. (total)**	**10**	**140**	**150**	**2.7**	**5–18**	**77**	**524**	**601**	**2.2**	**4**	**2–30**
*Campylobacter coli*	0	3	3	0.1	0–1	0	10	10	0.04	3	2–4
*Campylobacter fetus*	1	0	1	0.0	0–1	5	0	5	0.02	5	5–5
*Campylobacter jejuni*	3	76	79	1.4	0–11	27	251	278	1.0	4	2–16
*Campylobacter* spp.	6	61	67	1.2	2–11	45	263	308	1.1	5	2–30
***Listeria monocytogenes***	**9**	**2**	**11**	**0.2**	**0–4**	**64**	**4**	**68**	**0.2**	**6**	**2–35**
***Salmonella* spp. (total)**	**30**	**158**	**188**	**3.3**	**4–22**	**2,257**	**1,066**	**3,323**	**12.0**	**18**	**1^c^–1,149**
*Salmonella* Enteritidis	10	47	57	1.0	1–9	343	431	774	2.8	14	2–195
*Salmonella* Typhimurium	7	18	25	0.4	0–6	513	255	768	2.8	31	2–100
Other *Salmonella* serotypes	10	10	20	0.4	0–4	1,377	93	1,470	5.3	74	15–1,149
*Salmonella* spp.	3	83	86	1.5	0–10	24	287	311	1.1	4	2–26
***Shigella* spp.**	**0**	**7**	**7**	**0.1**	**0–2**	**0**	**203**	**203**	**0.7**	**29**	**2–162**
**STEC**	**5**	**6**	**11**	**0.2**	**0–2**	**82**	**21**	**103**	**0.4**	**9**	**2–41**
**Hepatitis A virus**	**5**	**5**	**10**	**0.2**	**0–2**	**62**	**36**	**98**	**0.4**	**10**	**3–15**
**Norovirus (total)**	**27**	**178**	**205**	**3.6**	**3–25**	**449**	**3,987**	**4,436**	**16.0**	**22**	**2–150**
Norovirus GI	4	12	16	0.3	0–3	96	345	441	1.6	28	2–74
Norovirus GI and GII	3	4	7	0.1	0–2	53	93	146	0.5	21	3–47
Norovirus GII	5	43	48	0.8	0–9	40	1,018	1,058	3.8	22	2–150
Norovirus	15	119	134	2.4	2–19	260	2,531	2,791	10.1	21	2–132
**Scombroid toxin/histamine**	**11**	**3**	**14**	**0.2**	**0–4**	**75**	**6**	**81**	**0.3**	**6**	**2–24**
**Other pathogens^a^**	**1**	**6**	**7**	**0.1**	**0–2**	**3**	**21**	**24**	**0.1**	**3**	**2–9**
**Two pathogens^b^**	**5**	**4**	**9**	**0.2**	**0–2**	**15**	**26**	**41**	**0.1**	**5**	**2–13**
All known	152	514	666	11.8	NA	3,565	5,967	9,532	34.4	14	1^c^–1,149
All unknown	NA	NA	4,991	88.2	NA	NA	NA	18,179	65.6	4	2–160

GI: genogroup I; GII: genogroup II; NA: not applicable; other: food vehicle not confirmed OR positive environmental swabs OR pathogen confirmed in case(s); STEC: Shiga toxin-producing *Escherichia coli*.

^a^ Other pathogens were found in a confirmed food vehicle (*Vibro parahaemolyticus*, one outbreak) or in other outbreaks including *Yersinia enterocolitica* (two outbreaks), rotavirus (one outbreak; positive environmental swab), *Giardia* (two outbreaks), and tapeworm (one outbreak).

^b^ Two pathogens were found in a confirmed food vehicle in five outbreaks: *B. cereus* and *C. perfringens* (two outbreaks), *B. cereus* and *S. aureus* (two outbreaks), and *B. cereus* and *S. Enteritidis* and for other in four outbreaks: *C. jejuni* and norovirus, *C. jejuni* and STEC, *S. Typhimurium* and STEC, *Dientamoeba fragilis* and *Blastocystis hominis.*

^c^ One case was an international outbreak with a single Dutch patient.

Rows in bold text indicate the subtotal for each particular pathogen or pathogen group. Rows that are not in bold are subgroups of a pathogen based on typing results.

**Figure 2:**
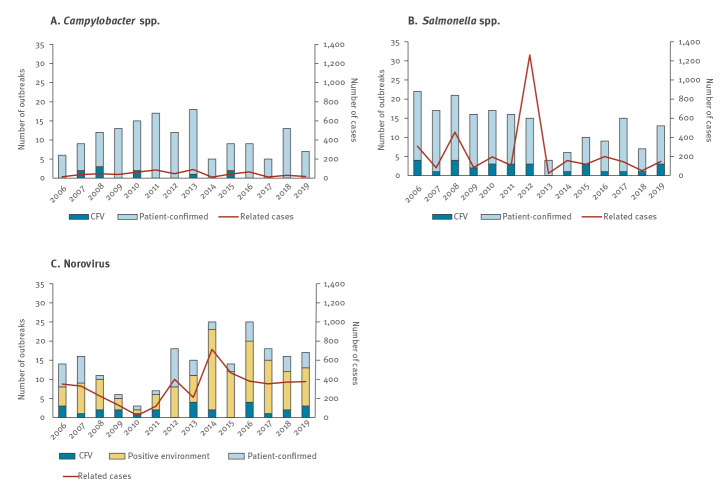
Number of food-borne outbreaks and related human cases per year for *Campylobacter* spp. (n = 150), *Salmonella* spp. (n = 188), and norovirus (n = 205), the Netherlands, 2006–2019

Norovirus caused most outbreaks and most related cases, followed by *Salmonella* spp. and *Campylobacter* spp (Figure 2). The mean number of cases per outbreak is lower for *Campylobacter* spp. (4 cases/outbreak) compared with norovirus (22 cases/outbreak) and *Salmonella* spp. (18 cases/outbreak). The three largest outbreaks all were caused by *Salmonella*: in 2012 due to *Salmonella* Thompson in smoked salmon with 1,149 reported cases [11], in 2006 due to *Salmonella* Typhimurium in cheese with 224 cases [12] and in 2008 due to *Salmonella* Enteritidis with 195 cases, most likely due to eggs although this was not confirmed. Recently, *Salmonella* outbreaks are less often seen; only four *Salmonella* outbreaks during 2013 and between six and 15 outbreaks in the years 2014–19 were observed, compared with between 15 and 22 outbreaks per year in 2006–12. *Campylobacter* outbreaks are less reported since 2014 compared with the period 2006–13. Reports of norovirus outbreaks have increased since 2012, primarily detected in environmental swabs.

CFV: confirmed food vehicle.

Patient-confirmed (all panels) indicates that the pathogen was confirmed only in case(s). Positive environment (yellow bars, panel C) indicate positive environmental swabs.

*B. cereus, C. perfringens*, and *S. aureus* are rarely diagnosed in cases, and almost only found in food. *B. cereus* is most often found in outbreaks with a confirmed food vehicle, followed by *Salmonella* spp. and norovirus (Table 1).

### Outbreak setting

Restaurants/deli/cafeteria were most frequently implicated in food-borne outbreaks (Table 2), and linked to 81.1% of not-confirmed outbreaks, followed by plant or facility (7.2%) and private home (5.0%). Of the confirmed food-borne outbreaks, restaurants/deli/cafeteria are mentioned in 59.2% of the outbreaks, followed by plant or facility (17.1%) and private home (6.6%). The restaurants/deli/cafeteria setting was found in 64.8% outbreaks with positive environmental swabs, followed by catering (17.2%) and institutions (6.3%); in outbreaks with confirmed cases, 32.4% were found in restaurants/deli/cafeteria, 27.5% in private home, and 17.6% were infected abroad.

**Table 2:** Number and percentage of food-borne outbreaks by place where food was prepared, and confirmation of pathogen, the Netherlands, 2006–2019 (n = 5,657)

Location	Confirmed food vehicle		Positive environmental swabs		Pathogen confirmed in case(s)		Pathogen not confirmed		Total
	n	%	n	%	n	%	n	%	
Restaurant/deli/cafeteria	90	2.1	83	1.9	125	2.9	4,046	93.1	4,344
Entertainment/party location	2	4.7	6	14.0	11	25.6	24	55.8	43
Catering	8	9.1	22	25.0	10	11.4	48	54.5	88
Fair/mobile service	1	0.9	0	0.0	1	0.9	104	98.1	106
Farm (shop)	7	58.3	0	0.0	1	8.3	4	33.3	12
Plant or facility	26	6.3	2	0.5	24	5.8	359	87.3	411
Institution	2	3.5	8	14.0	13	22.8	34	59.6	57
Private home	10	2.7	0	0.0	106	29.1	248	68.1	364
Infected abroad	2	2.8	0	0.0	68	95.8	1	1.4	71
Other/unknown	4	2.5	7	4.3	27	16.8	123	76.4	161
Total	152	2.7	128	2.3	386	6.8	4,991	88.2	5,657

### Food products

Among the 152 outbreaks with a confirmed food vehicle, a total of 165 food products tested positive; in seven outbreaks, two food products tested positive and in three outbreaks three food products were positive. In nine out of these 10 outbreaks, the pathogens were a combination of *B. cereus, C. perfringens*, and/or *S. aureus*, and in the tenth outbreak, poultry tested positive for *Salmonella* Enteritidis and a composed product was positive for *B. cereus*. Furthermore, two food products (composed products) tested positive for both *B. cereus and S. aureus*.

Overall, red meat (n = 31) and composed products (n = 30) were the most common contaminated food products (Table 3). The pathogens most commonly identified in outbreaks caused by composed products, cereals/pasta/rice and, to a lesser degree red meat, were *B. cereus, C. perfringens*, and *S. aureus*. Other common pathogen–food pairs identified were *Campylobacter*–dairy, *Salmonella*–red meat, *Salmonella* Enteritidis–eggs, hepatitis A virus–fruit/vegetables, norovirus–shellfish, and scombroid toxin–fish (tuna in 10/11 outbreaks).

**Table 3:** Number of contaminated food products found in outbreaks with a confirmed food vehicle, by aetiology and food commodity, the Netherlands, 2006–2019 (n = 165)

Pathogen	Fish	Shellfish	Red meat^a^	Poultry	Eggs	Dairy	Fruit/ vegetables	Cereals/ pasta/rice	Pastry/ cookies	Soup/ sauce	Composed Product^b^	Total^c^
***Bacillus cereus***	**0**	**1**	**6**	**1**	**2**	**1**	**1**	**10**	**1**	**3**	**16**	**42**
***Clostridium perfringens***	**0**	**0**	**2**	**3**	**0**	**0**	**1**	**0**	**0**	**0**	**5**	**11**
***Staphylococcus aureus***	**0**	**0**	**5**	**0**	**0**	**0**	**1**	**4**	**0**	**0**	**1**	**11**
***B.cereus* and *S. aureus***	**0**	**0**	**0**	**0**	**0**	**0**	**0**	**0**	**0**	**0**	**2**	**2**
***Campylobacter* spp. (total)**	**0**	**0**	**1**	**3**	**0**	**5**	**0**	**0**	**0**	**0**	**1**	**10**
*Campylobacter fetus*	0	0	0	0	0	1	0	0	0	0	0	1
*Campylobacter jejuni*	0	0	0	0	0	3	0	0	0	0	0	3
*Campylobacter* spp.	0	0	1	3	0	1	0	0	0	0	1	6
***Listeria monocytogenes***	**3**	**1**	**2**	**2**	**0**	**0**	**0**	**1**	**0**	**0**	**0**	**9**
***Salmonella* spp. (total)**	**1**	**1**	**12**	**5**	**6**	**2**	**2**	**0**	**0**	**0**	**2**	**31**
*Salmonella* Enteritidis	0	1	3	1	6	0	0	0	0	0	0	11
*Salmonella* Typhimurium	0	0	5	0	0	2	0	0	0	0	0	7
Other *Salmonella* serotypes	1	0	3	3	0	0	2	0	0	0	1	10
*Salmonella* spp.	0	0	1	1	0	0	0	0	0	0	1	3
**STEC (total)**	**0**	**0**	**3**	**0**	**0**	**0**	**2**	**0**	**0**	**0**	**0**	**5**
STEC O104:H4	0	0	0	0	0	0	1	0	0	0	0	1
STEC O157	0	0	3	0	0	0	1	0	0	0	0	4
***Vibrio parahaemolyticus***	**0**	**1**	**0**	**0**	**0**	**0**	**0**	**0**	**0**	**0**	**0**	**1**
**Hepatitis A virus**	**0**	**1**	**0**	**0**	**0**	**0**	**4**	**0**	**0**	**0**	**0**	**5**
**Norovirus (total)**	**0**	**21**	**0**	**0**	**0**	**0**	**1**	**0**	**2**	**0**	**3**	**27**
Norovirus GI	0	2	0	0	0	0	0	0	1	0	1	4
Norovirus GI and GII	0	3	0	0	0	0	0	0	0	0	0	3
Norovirus GII	0	4	0	0	0	0	0	0	0	0	1	5
Norovirus	0	12	0	0	0	0	1	0	1	0	1	15
**Scombroid toxin/histamine**	**11**	**0**	**0**	**0**	**0**	**0**	**0**	**0**	**0**	**0**	**0**	**11**
Total	15	26	31	14	8	8	12	15	3	3	30	165

GI: Genogroup I; GII: Genogroup II; STEC: Shiga toxin-producing *E. coli*.

**^a^** Red meat is beef and/or pork.

**^b^** Composed products are dishes with a mixture of ingredients, for example vegetables, meat, and/or rice.

^c^ In seven outbreaks, two food products tested positive and in three outbreaks, three food products.

Rows in bold text indicate the subtotal for each particular pathogen or pathogen group. Rows that are not in bold are subgroups of a pathogen based on typing results.

## Discussion

Twelve years of data from three surveillance pathways was utilised for a long-term descriptive analysis of food-borne outbreaks in the Netherlands. The majority of the outbreaks were registered at the NVWA by citizens. A much lower number of outbreaks were notified by PHS; however, since those reporting the outbreak were health care professionals, such as doctors and microbiologists, the chance of detecting a pathogen was much larger. A sizeable increase in reported outbreaks has been observed since 2015, when reporting criteria were expanded to include outbreaks registered by the NVWA in which no food samples were taken. Although criteria were widened to reduce the under-reporting, the details of these additional outbreaks were limited, especially when the sources were unconfirmed. 

Exploring trends per pathogen was not affected by the changes in reporting criteria, although only the annual number of outbreaks caused by norovirus, *Salmonella* and *Campylobacter* were high enough to be analysed. The emphasis for the registration and analytic developments of virus outbreaks from 2012 onwards led to an increase of reports of norovirus outbreaks, especially those with positive swabs. The largest food-borne outbreak recorded in the Netherlands occurred in 2012, caused by *Salmonella* Thompson in smoked salmon [11]. Based on the 1,149 reported cases, it was calculated that more than 21,000 people had been infected, with an estimated total outbreak cost of EUR 7.5 million of which EUR 6.8 million was the cost-of-illness [13]. Strikingly, after this large outbreak, the number of *Salmonella* outbreaks dropped significantly without an obvious reason. Up to 2012, a decrease in cases was also seen in the sentinel *Salmonella* laboratory surveillance, but followed by a stabilisation afterwards [14]. For *Campylobacter,* a decrease in cases in the sentinel laboratory surveillance was observed since 2014, although this decrease seemed more prominent in the number of outbreaks than in the total number of cases [14].

A large proportion of the norovirus outbreaks were identified by positive environmental swabs, supported by confirmed norovirus in cases or reported symptoms pointing towards norovirus. Presence of norovirus on utensils and surfaces can point to an infected food handler who has contaminated food during preparation caused by flaws in hygiene measures [15]. Nevertheless, person-to-person transmission cannot be ruled out in these cases. In the confirmed food vehicle outbreaks of norovirus, 21 of 27 outbreaks were related to shellfish. The point of contamination of shellfish with viruses is most likely in the area where the seafood is farmed on account of sewage inlet [15]. The other six norovirus outbreaks with a confirmed food vehicle were more likely to have been caused by infected food handlers. *Salmonella* outbreaks with a confirmed food vehicle were most likely related to red meat, eggs (all *S*. Enteritidis), poultry meat and dairy, consistent with the main reservoirs for *Salmonella* and as reported in food-borne outbreak overviews of other countries worldwide [6,16-18]. A notable difference between *Campylobacter* versus *Salmonella* and norovirus was the number of cases per outbreak, which was much lower in *Campylobacter* outbreaks. Similar differences in number of cases per outbreak for these pathogens were reported in New South Wales, Australia and the United States [6,18], whereas in the United Kingdom, the mean number of cases per outbreak did not differ significantly for *Salmonella* and *Campylobacter* [17]. An explanation for the smaller outbreaks could be the lack of growth of *Campylobacter* in food, in contrast to *Salmonella* [19]. Only in a small part of the *Campylobacter* outbreaks could a food product be confirmed, in which dairy (raw milk/raw milk cheese) and poultry were most prominent. An attribution study estimated that 66% of the human *Campylobacter* infections in the Netherlands originated from chicken, 21% from cattle, 3% from sheep, and 0.3% from pigs [20]. In the United States, England and Wales, where larger numbers of *Campylobacter* outbreaks were recorded, poultry, dairy, water and composed foods were the most relevant sources [6,17].

The toxin-producing bacteria *S. aureus*, *C. perfringens* and, in particular, *B. cereus* were other main pathogens in the confirmed food-borne outbreaks. These bacteria were detected in all food groups, except fish, but with a preference for composed food and cereals/pasta/rice. *B. cereus* is ubiquitously present in the environment and food products, *S. aureus* is typically found on skin and in the nasal passage and *C. perfringens* exists in soil, intestinal flora and animals [21-24]. Inadequate temperature management, such as slow or inadequate cooling and storage of food products at elevated temperatures between 10°C and 60°C, may allow multiplication of the bacteria and production of toxins [21,22,25]. Poor hygiene and improper cleaning could introduce contamination of products at any stage from primary production up to serving the food. The number of viable *B. cereus* cells can be reduced when a product has been reheated, eliminating the bacteria [25]. However, toxins already present before reheating will remain and can cause disease. Although the majority of food-borne outbreaks caused by *B. cereus* show concentrations above 100,000 cfu/g in the contaminated product, outbreaks with concentrations between 1,000 and 100,000 cfu/g have also been reported [26]. In the present analyses, outbreaks with counts below 100,000 cfu/g for *B. cereus*, *C. perfringens* or *S. aureus* were classified as ‘unknown causative agent’. This could have led to an underestimation of outbreaks caused by these toxin-producing bacteria, as another 55 outbreaks were within the range 1,000 and 100,000 cfu/g; this includes 39 outbreaks in which *B. cereus* was found in a food product, six outbreaks with *C. perfringens*, eight with *S. aureus*, and two with *B. cereus* and either *C. perfringens* or *S. aureus* (data not shown). Disease on account of these toxin-producing bacteria, which is symptomatically similar, was rarely confirmed in cases, as it is mostly short and self-limiting [21,27,28]. This causes a diagnostic deficit, as cases will rarely seek medical attention and no laboratory analyses are performed.

The majority of the outbreaks were registered at the NVWA, and mainly have restaurants/delis/cafeteria as the setting. This could indicate that, during an inspection by the food safety authority, inspectors should check whether the relevant requirements for food handling and preparation are met. On the other hand, these outbreaks are mainly reported by citizens and were often not pathogen-confirmed. In outbreaks in which the pathogen was confirmed in the cases, our data indicates that the overall percentage of public eating places was lower and that private homes were also a setting for outbreaks. A tendency to detect or report an outbreak in a public eating place more frequently as compared with home can, therefore, not be ruled out.

Contamination of food can occur at any point from farm to table. The outbreaks with positive environmental swabs and those caused by the three toxin-producing bacteria, mainly found in composed products and cereals/pasta/rice, were most likely the result of improper hygiene, handling, storage and preparation in the last stage before consumption. Shellfish (norovirus, hepatitis A virus), red meat (*Salmonella*, STEC), eggs (*Salmonella*), dairy (*Campylobacter*, *Salmonella*) and fruit/vegetables (*Salmonella*, STEC, hepatitis A virus) are products with highest risk of being contaminated during the production process. The risk of people becoming infected arises when these products are eaten raw or undercooked, as thorough heating would reduce or eliminate the pathogens. High production standards including control measures must be in place to reduce the risk. Even when some foods, e.g. red meat or poultry, are eaten after thorough heating, improper food handling during preparation could lead to cross contamination and thus illness [29]. However, in our study, the moment of contamination could not be analysed with the current information.

Furthermore, the pathogen causing the disease remained unknown in the majority of the outbreaks (88.2%), consistent with observations from New South Wales, Australia [18]. Several factors contribute to this gap. Firstly, most pathogens have an incubation period of at least several days [30]. Often, leftovers of the implicated food items will no longer be available for testing when disease symptoms arise and are reported. This is amplified when reporting is done after the pathogen is detected in cases, since analysis can take several days. The probability that the batch of the implicated food item is no longer available increases with an increasing time window. Furthermore, the incubation period introduces recall bias, which could lead to incorrect designation of a certain food item as the possible contaminated food product, especially in small outbreaks with cases, i.e. members of one household, that share more than one common food item. This is exacerbated by the inclusion of outbreaks reported at the NVWA directly by citizens. Another possibility is that food was implied as source of the infection, but in reality was caused by person-to-person spread.

Food is produced and transported globally. When a food product is contaminated early in the food production chain, cases can spread over a large geographical area. A national surveillance system including sequence data of human isolates offers the opportunity to follow the different strains seen within the country and detect even small clusters that spread nationwide. Such pathogen-based laboratory surveillance systems are developed for *Salmonella*, *L. monocytogenes*, STEC and hepatitis A virus in the Netherlands, which has led to the identification of several national outbreaks that otherwise would not have been seen at all or not caught until a much later stage. An isolate-based surveillance system is currently developed for *Campylobacter*, which shows a stable high incidence as well as disease burden, indicating that efforts to reduce this pathogen at primary production or during the handling and processing phase is not effective. Identifying national clusters increases the opportunity for case–control studies and source tracing by which potential sources can be identified and possibly be eliminated, thereby reducing the disease burden. In addition, the national system currently in place also opens the opportunity to communicate with other, surrounding countries to determine whether an outbreak is seen internationally. In some cases, this can even lead to a joint investigation, as occurred with several outbreaks in our overview, e.g. STEC in lettuce together with Iceland [31], *Salmonella* Newport in bean sprouts together with Germany [32], and *Salmonella* Enteritidis in eggs in a joint European investigation [33].

## Conclusions

Usage of different pathways to monitor food-borne outbreaks aids insight in the occurrence of these outbreaks. PHS register local outbreaks, national disease-specific surveillance systems detect outbreaks dispersed over a larger area, and citizens report outbreaks that otherwise would have been missed. Analysis of 12 years of food-borne outbreaks reveals the persistent occurrence of these outbreaks in the Netherlands. A multiplicity of pathogens can cause illness via a vast array of food products, complicating control and elimination of food-borne illness. Contamination during the production process, consumption of raw products, and improper hygiene, handling, storage and preparation are the main underlying factors that could be addressed to reduce the number of food-borne outbreaks. Overall, norovirus, *Salmonella* and *Campylobacter* appeared to be the most prevalent pathogens related to food-borne outbreaks. Additionally, specific connections were seen between pathogens and food commodities. These results will guide in future outbreak investigations to focus the search to the source where possible. 
